# Salmon and human calcitonin like material in lung cancer.

**DOI:** 10.1038/bjc.1985.136

**Published:** 1985-06

**Authors:** C. Gropp, W. Luster, K. Havemann


*Br. J. Cancer* (1985), **51**, 897–901

### Short Communication

### Salmon and human calcitonin like material in lung cancer

C. Gropp, W. Luster & K. Havemann

*Department of Hematology/Oncology – University of Marburg, FRG.*

Human calcitonin (hCT) like material has been detected in plasma, urine and tissue extracts of patients with medullary thyroid carcinoma (MTC) and lung cancer as well as in the incubation medium of cultured cells of these tumours (Bertagna *et al.*, 1978; Ellison *et al.*, 1975; Goltzman & Tischler, 1978; Goltzman *et al.*, 1974; Gropp *et al.*, 1980*a,b*; Ham *et al.*, 1980; Luster *et al.*, 1982; Milhaud *et al.*, 1974; Silva *et al.*, 1974; Sizemore & Heath, 1975). The hCT immunoreactive material has been shown to be useful as tumour markers in patients with MTC and with small cell lung cancer (SCLC) to determine prognosis and response to therapy (Goltzman *et al.*, 1974; Gropp *et al.*, 1980*a,b*). The hCT has not only been detected in man but also in protochordates (Galan *et al.*, 1981*a*, Girgis *et al.*, 1980), in amphibia (Galan Galan *et al.*, 1981*b*, Perez Cano *et al.*, 1981) and in birds (Perez Cano *et al.*, 1982). In these animals hCT coexists with other calcitonins like salmon calcitonin (sCT). Recently the existence of sCT in human thyroid and brain has been described by Fischer *et al.*, 1983. This study shows for the first time the coexistence of both hCT- and sCT-like material in sera of patients with SCLC and in the culture medium of established lung cancer cell lines.

hCT and sCT were determined simultaneously in about 100 patients with histologically proven SCLC before therapy. Prior to therapy, extent of disease was determined by physical examination, liver function tests, chest and bone X-rays, broncho- and/or mediastinoscopy, bone marrow examination, ultrasound, computerized tomography and nuclear scans of the bone. Patients were divided into limited and extensive categories. Limited disease implied that the disease was confined to one hemithorax with or without mediastinal node disease, with or without ipsilateral supraclavicular node involvement and with or without ipsilateral small pleural effusion (without malignant cells). Extensive disease was defined as disease beyond the confines of the definition of limited disease. Both calcitonins were also assayed in the medium of lung tumour cell lines established in our laboratory (Gropp *et al.*, 1984, Luster *et al.*, 1984). For comparison, the culture medium of our medullary carcinoma cell lines were also studied as well as 18 sera of patients with medullary carcinoma of the thyroid. Permanent cell cultures were established from small cell, large cell, squamous and adeno carcinoma of the lung. All purification steps leading to a permanent cell line were controlled by cytological analysis. Cell lines of small cell carcinoma were derived from pleural or pericardial exudates. Specimens were collected with an anticoagulant, centrifuged and then separated from erythrocytes and cell debris by ficoll gradient (Pharmacia Uppsala, Sweden) centrifugation 30 min at 800 *g*. The fraction containing the tumour cells was carefully collected and washed in a 10-fold volume of MEM Dulbecco's or RPMI-1640 cell culture medium (Boehringer Mannheim, FRG). The cells were resuspended in the same medium containing 16.6% of foetal calf serum (Paesel Frankfurt, FRG), diluted to $10^5$ cells $ml^{-1}$ and plated into cell culture flasks (Nunc Roskilde, Denmark). Non small cell lung tumour cell lines were established from surgically obtained fresh tumour tissue. Solid tissue specimens were washed with antibiotics (0.5 g streptomycin, 50,000 i.u. penicillin and 250 $\mu$g amphotericin B $ml^{-1}$ PBS) and minced into 1–3 $mm^3$ pieces. To obtain a cell suspension the material was disintegrated by collagenase (0.5 mg $ml^{-1}$ PBS, Boehringer Mannheim, FRG) 3 times for 15 min at 20°C. In the case of an incomplete disintegration after this procedure an incubation in the presence of 0.5 mg $ml^{-1}$ collagenase and 0.5% trypsin (Boehringer Mannheim, FRG) was followed. Washing and culture of the tumour cells was performed as described above for the small cell tissue cultures.

After growth of the cells to confluency or to 1 to $5 \times 10^6$ cells per culture flask the medium was analyzed for peptide hormone content. The positive cell cultures were cloned in microplates, analyzed for peptide hormones and recloned. If necessary such clones were submitted to an additional purification by soft agar cloning.

---

Correspondence: C. Gropp, Abt. Hämatologie/Onkologie, Zentrum f. Innere Medizin, Baldingerstrasse, D-3550 Marburg/L.
Received 5 January 1985; and in revised form 20 February 1985

© The Macmillan Press Ltd., 1985

hCT was determined by means of a commercial radioimmunoassay using an antiserum against synthetic hCT (1–32) as described previously (Luster et al., 1982). sCT was determined by a radioimmunoassay using an antiserum against sCT (16–32) which showed no cross reactivity against hCT. The double antibody radioimmunoassay was obtained from Diagnostik System Laboratories (Webster, Texas) and is not available commercially.

For further characterization of the hCT- and sCT-like material the incubation medium of two small cell lung cancer cell lines was subjected to gel chromatography and the fractions were assayed simultaneously for hCT and sCT. For this study 3 ml samples of culture medium were subjected to gel filtration on a 1.5 × 90 cm column of ACA 54 (LKB Stockholm, Sweden), equilibrated with 80 mM $KH_2PO_4$, 0.4 M EDTA and 0.1% $NaN_3$, pH 7.4, or with the same buffer containing proteinase inhibitors. Protein was eluted in the presence of the above buffers under a flow rate of 8 ml $h^{-1}$. Four ml fractions were collected, lyophilized and resuspended in 400 $\mu$l of distilled water in order to estimate calcitonin. The column was calibrated with blue dextran (Pharmacia, Uppsala, Sweden), aldolase (Boehringer Mannheim, FRG), bovine serum albumin, ovalbumin, chymotrypsinogen A (Pharmacia, Uppsala, Sweden), myoglobin whale, cytochrome, c, cyanocobalamin, bromophenol-blue (Serva, Heidelberg, FRG) and [$^{125}$I]-calcitonin (Immunonuclear, Stillwater, MN).

hCT-immunoreactive material was elevated (> 100 pg $ml^{-1}$) in 20/101 (20%) patients with SCLC compared to age and sex matched controls. In 54 (53%) of these patients elevated sCT-immunoreactive material (> 1 ng $ml^{-1}$) was found (Figure 1*a*). There was no correlation between the calcitonins in patients sera. As shown in Figure 1*b*, elevated sCT immunoreactivity was seen in patients with low as well as with high hCT immunoreactivity.

Concerning the stage of disease hCT-immunoreactive material was elevated in 18% of patients with limited disease and in 34% of patients with extensive disease. For sCT-like material the frequency was 33% (limited disease) and 40% (extensive disease) (Figure 2).

High amounts of the sCT-like material could be detected in the incubation medium of several lung cancer cell lines (Figure 3).

In the medium of the lung cancer cell lines the sCT immunoreactivity was higher than the hCT-immunoreactivity. In contrast, in the medium of the medullary carcinoma cell lines hCT-immunoreactivity was higher than the sCT-immunoreactivity. The same trend was found in sera of 18 patients with medullary carcinoma. Here hCT was elevated in 72%, whereas sCT was only elevated in 39%.

The chromatography results show the presence of three immunoreactive forms of hCT with mol. w of 100,000, 48,000 and 20,000 daltons, all larger than the physiological hCT. These results have been described previously (Luster et al., 1982). The simultaneous determination of sCT immunoreactivity detected four sCT immunoreactive fractions with mol. w of 100,000, 25,000, 13,000 and 5,000 (Figure 4).

Our results show for the first time the coexistence of elevated values of hCT- and sCT-like material in the serum of patients with SCLC. It could be demonstrated that both calcitonin immunoreactivities are secreted into the incubation medium by cultured lung cancer cells.

Here, in contrast to studies with medullary carcinoma cells, the values for sCT-like material are higher than for the hCT-like material. The same results were found *in vivo* where in lung cancer patients the sCT values were higher than the hCT values and in medullary cancer patients vice versa.

Our studies also confirm reports from Fischer et al. (1983) who demonstrated both hCT and sCT in human thyroid and brain. From our studies the nature of the sCT-like material is not clear. From recent investigations of the hCT in lung and medullary cancer patients and from the chromatography studies it can be suggested that we detected by radioimmunoassay an sCT precursor molecule from which the sCT may derive. For hCT-like material high molecular precursor forms with a mol. w in the range from 8,000–55,000 daltons have been isolated from lung and medullary carcinomas (Luster et al., 1982, Jacobs et al., 1981; Desplan et al., 1980; Roos et al., 1980; Allison et al., 1981). In any case the sCT-like material may be of further interest as a tumour marker in lung cancer patients. Compared to other tumour markers, the incidence of sCT-immunoreactive material was similar to carcinoembryonic antigen (CEA) and neuron specific enolase (NSE) in patients with SCLC. In this tumour elevated CEA levels have been found in ~50% (Concannon et al., 1974, Vincent et al., 1973, Gropp et al., 1978) and elevated NSE levels in 45–69% (Gropp et al., unpublished, Carney et al., 1982). Whereas elevated CEA and NSE values were mostly observed in patients with extensive disease there was no statistical difference for the frequency of sCT-like material in patients with limited or extensive SCLC. So sCT-determination probably may be used in the future concomitantly with other tumour markers in patients with SCLC. In addition, further studies for the biochemical characterization of the higher mol. wt sCT-fractions are on the way.

**Figure 1a** Salmon and human calcitonin serum values in patients with small cell lung cancer.

**Figure 1b** Salmon calcitonin serum values in patients with small cell lung cancer and low (i) and high (ii) human calcitonin values.

**Figure 2** Salmon and human calcitonin values in patients with small cell lung cancer staged as limited and extensive disease.

**Figure 3** Salmon (▲) and human (●) calcitonin values, measured simultaneously in the culture medium of different permanent lung cancer cell lines of various histology and in cell lines from medullary carcinomas of the thyroid.

**Figure 4** AcA54 column chromatography of the culture medium of a small cell lung cancer cell line. Fractions were assayed for salmon (▲) and human (●) calcitonin.

**References**

ALLISON, J., HALL, L., MACINTYRE, I. & CRAIG, R.K. (1981). The construction and partial characterization of plasmids containing complementary DNA sequences to human calcitonin precursor polyprotein. *Biochem. J.*, **199**, 725.

BERTAGNA, X.Y., NICHOLSON, W.E., PETTENGILL, O.S., SORENSEN, G.D., MOUNT, C.D. & ORTH, D.N. (1978). Ectopic production of high molecular weight calcitonin and corticotropin by human cell carcinoma cells in tissue culture. *J. Clin. Endocrinol. Metab.*, **47**, 1390.

CARNEY, D.N., IHDE, D.C., COHEN, M.H. & 4 others. (1982). Serum neuron-specific enolase: A marker for disease extent and response to therapy of small-cell lung cancer. *Lancet*, **i**, 583.

CONCANNON, J.P., DALBOW, M.H., LIEBLER, G.A., BLAKE, K.E., WEIL, C.S. & COPPER, J.W. (1974). The carcinoembryonic antigen assay in bronchogenic carcinoma. *Cancer*, **34**, 184.

DESPLAN, C., BENICOURT, C., JULLIENNE, A. & 4 others. (1980). Cell-free translation of mRNS coding for human and murine calcitonin. *FEBS Lett.*, **117**, 89.

ELLISON, M.L., WOODHOUSE, D., HILLYARD, C. & 5 others. (1975). Immunoreactive calcitonin production by human lung carcinoma cells in culture. *Br. J. Cancer*, **32**, 373.

FISCHER, J.A., TOBLER, P.H., HENKE, H. & TSCHOPP, F.A. (1983). Salmon and human calcitonin-like peptides coexist in the human thyroid and brain. *J. Clin. Endocrinol. Metab.*, **57**, 1314.

GALAN GALAN, F., ROGERS, R.M., GIRGIS, S.I. & 4 others (1981*a*). Immunochemical characterization and distribution of calcitonin in the lizard. *Acta Endocrinol.*, **97**, 427.

GALAN GALAN, F., ROGERS, R.M., GIRGIS, S.I. & MACINTYRE, I. (1981*b*). Immunoreactive calcitonin in the central nervous system of the pigeon. *Brain Res.*, **212**, 59.

GIRGIS, S.I., GALAN GALAN, F., ARNETT, T.R. & 4 others. (1980). Immunoreactive human calcitonin-like molecule in the nervous systems of protochodates and a cyclostome, Myxine. *J. Endocrinol.*, **87**, 375.

GOLTZMAN, D. & TISCHLER, A.S. (1978). Characterization of calcitonin released by medullary thyroid carcinoma in tissue culture. *J. Clin. Invest.*, **61**, 449.

GOLTZMAN, D., POTTS, J.T., JR., RIDGWAY, E.D. & MALOOF, F. (1974). Calcitonin as a tumor marker: Use of the radioimmunoassay for calcitonin in the postoperative evaluation of patients with medullary thyroid carcinoma. *N. Engl. J. Med.*, **290**, 1035.

GROPP, C., HAVEMANN, K. & LEHMANN, F.-G. (1978). Carcinoembryonic antigen and ferritin in patients with lung cancer before and during therapy. *Cancer*, **42**, 2802.

GROPP, C., HAVEMANN, K., SCHEUER, A. (1980*a*). Ectopic hormones in lung cancer patients at diagnosis and during therapy. *Cancer*, **46**, 347.

GROPP, C., HAVEMANN, K., PFLÜGER, K.H. (1980*b*). Calcitonin als Tumormarker beim Bronchialkarzinom. *Dtsch. Med. Wschr.*, **105**, 1175.

GROPP, C., LUSTER, W., HAVEMANN, K., WAHL, R. & RÖHER, H.D. (1984). Lung and gastrointestinal tumor cells secrete peptide hormones. *Protides Biol. Fluid*, **31**, 599.

HAM, J. ELLISON, M.L. & LUMSDEN, J. (1980). Tumor calcitonin, interaction with specific calcitonin receptors. *Biochem. J.*, **190**, 545.

JACOBS, J.W., LUND, P.K., POTTS, J.T., JR, BELL, N.H. & HABENER, J.F. (1981). Procalcitonin is a glycoprotein. *J. Biol. Chem.*, **256**, 2803.

LUSTER, W., GROPP, C., SOSTMANN, H., KALBFLEISCH, H. & HAVEMANN, K. (1982). Demonstration of immunoreactive calcitonin in sera and tissues of lung cancer patients. *Eur. J. Cancer Clin. Oncol.*, **18**, 1275.

LUSTER, W., GROPP, C., KERN, H.F. & HAVEMANN, K. (1984). Biosynthesis of hormone immunoreactive proteins by non-small cell lung tumor cells *in vitro*. *Acta Endocrinol.*, **105**, (Suppl. 264).

MILHAUD, G., CALMETTE, C., TABOULET, J., JULLIENNE, A. & MOUKHTAR, M.S. (1974). Hypersecretion of calcitonin in neoplastic conditions. *Lancet*, **i**, 462.

PEREZ CANO, R., GALAN GALAN, F., GIRGIS, S.I., ARNETT, T.R. & MACINTYRE, I. (1981). A human calcitonin-like molecule in the ultimobranchial body of the amphibia (Rana pipiens). *Experientia*, **37**, 1116.

PEREZ CANO, R., GIRGIS, S.I., GALAN GALAN, F. & MACINTYRE, I. (1982). Identification of both human and salmon calcitonin-like molecules in birds suggesting the existence of two calcitonin genes. *J. Endocrinal.*, **92**, 351.

ROOS, B.A., LINDALL, A.W., BAYLIN, S.M. & 4 others. (1980). Plasma immunoreactive calcitonin in lung cancer. *J. Clin. Endocrinol. Metab.*, **50**, 659.

SILVA, O.L., BECKER, K.L., PRIMACK, A., DOPPMANN, I. & SNIDER, R.H. (1974). Ectopic section of calcitonin by oat-cell carcinoma. *N. Engl. J. Med.*, **290**, 1122.

SIZEMORE, G.W. & HEATH, H. (1975). Immunochemical heterogeneity of calcitonin in plasma of patient with medullary thyroid carcinoma. *J. Clin. Invest.*, **55**, 1111.

VINCENT, R.G. & CHU, T.M. (1973). Carcinoembryonic antigen in patients with carcinoma of the lung. *J. Thor. Cardiovasc. Surg.*, **66**, 320.


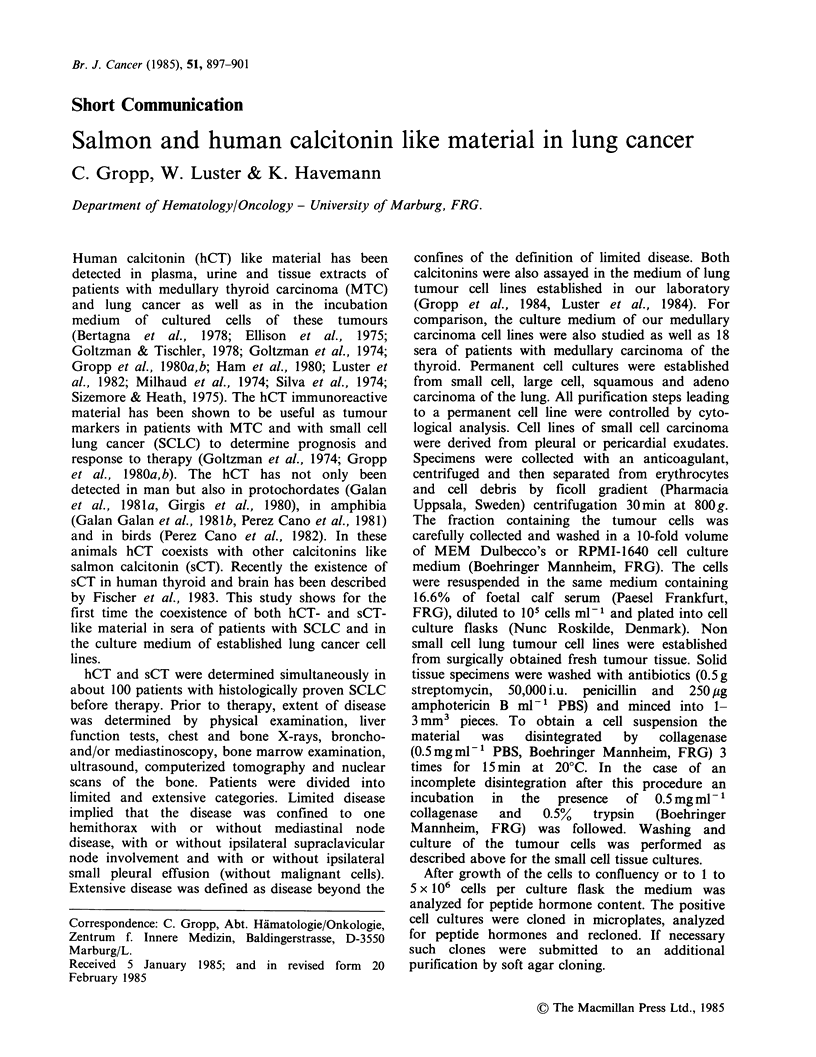

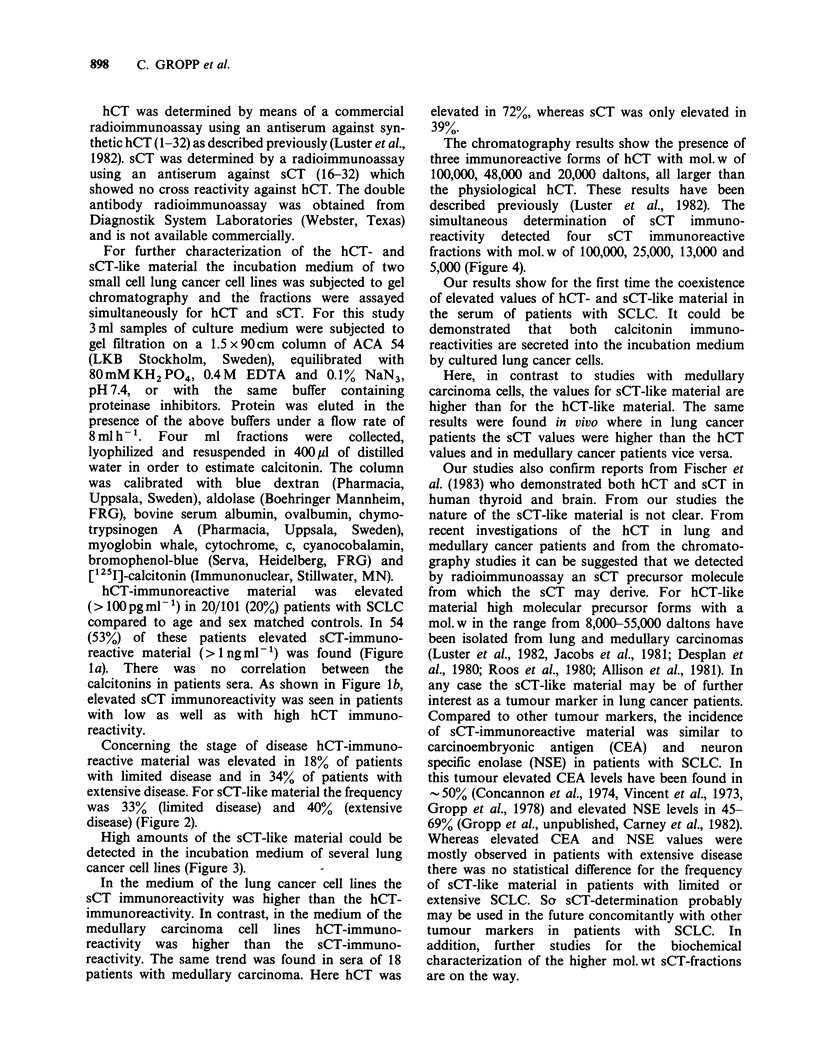

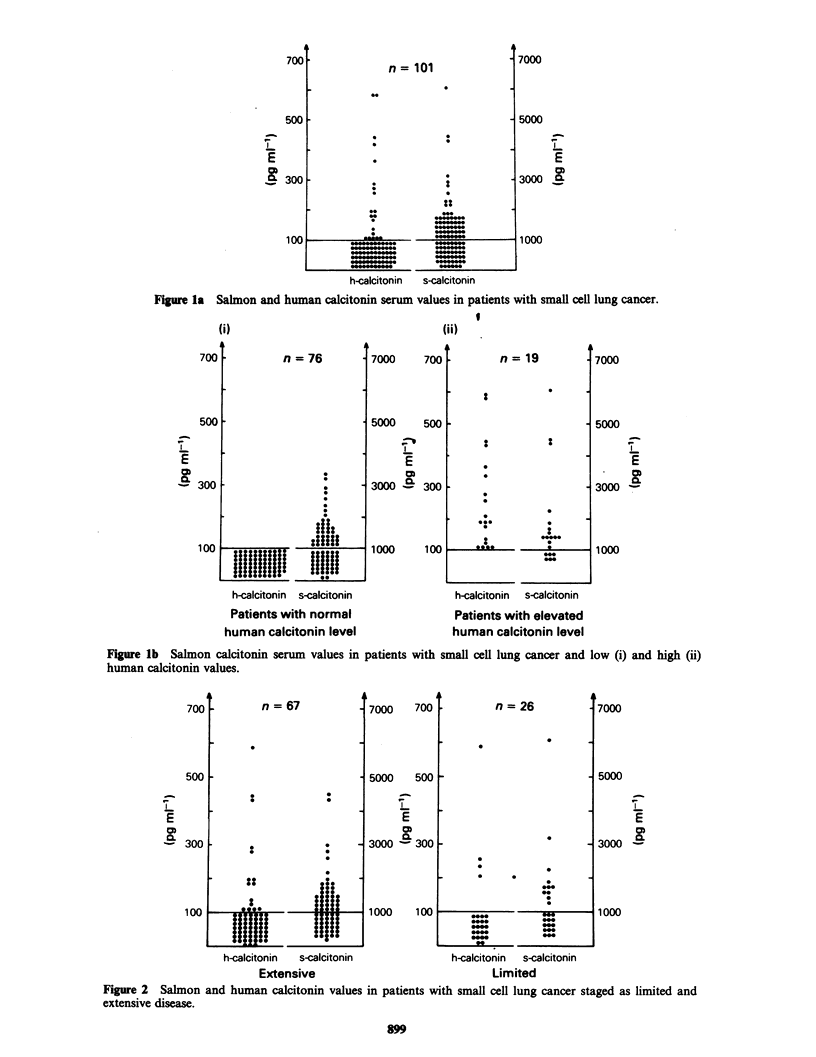

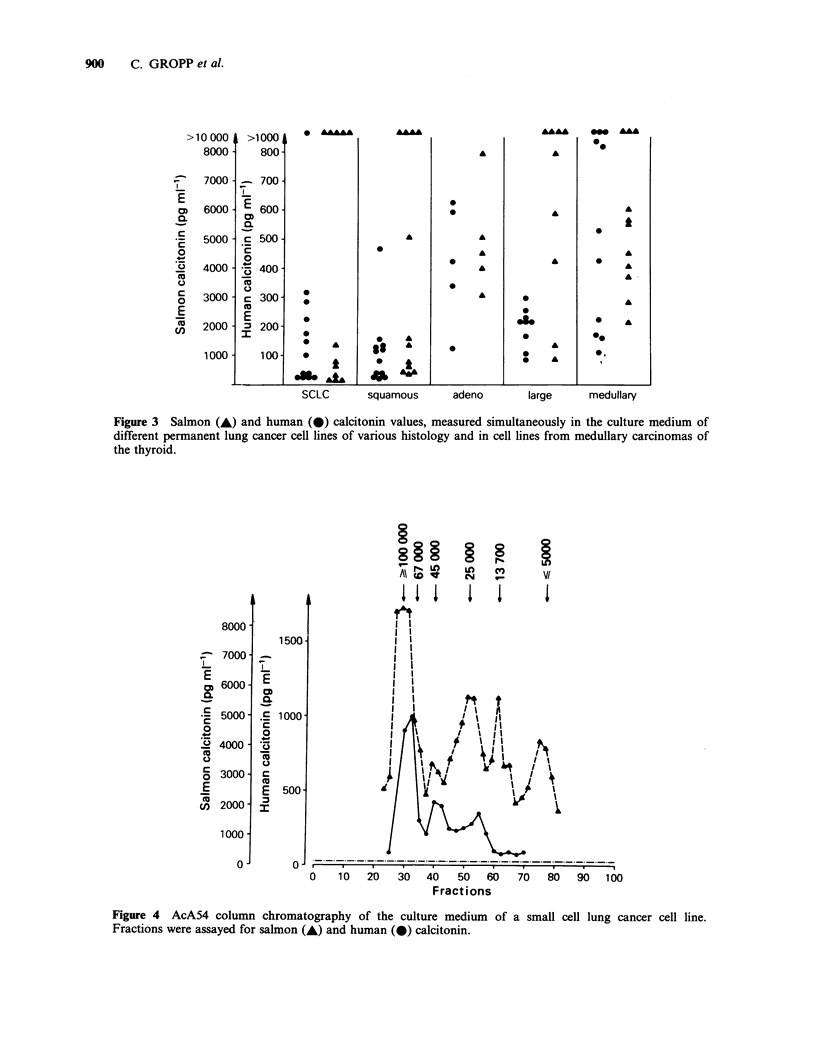

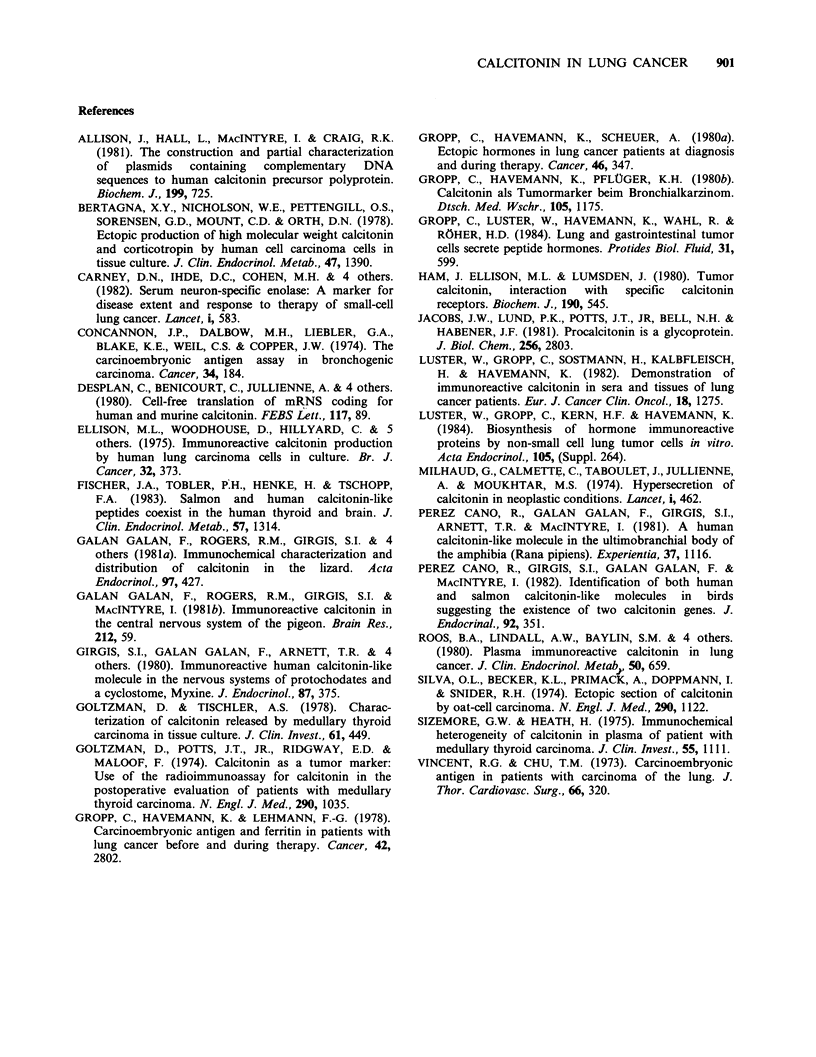


## References

[OCR_00588] Allison J., Hall L., MacIntyre I., Craig R. K. (1981). The construction and partial characterization of plasmids containing complementary DNA sequences to human calcitonin precursor polyprotein.. Biochem J.

[OCR_00595] Bertagna X. Y., Nicholson W. E., Pettengill O. S., Sorenson G. D., Mount C. D., Orth D. N. (1978). Ectopic production of high molecular weight calcitonin and corticotropin by human small cell carcinoma cells in tissue culture: evidence for separate precursors.. J Clin Endocrinol Metab.

[OCR_00602] Carney D. N., Marangos P. J., Ihde D. C., Bunn P. A., Cohen M. H., Minna J. D., Gazdar A. F. (1982). Serum neuron-specific enolase: a marker for disease extent and response to therapy of small-cell lung cancer.. Lancet.

[OCR_00608] Concannon J. P., Dalbow M. H., Liebler G. A., Blake K. E., Weil C. S., Cooper J. W. (1974). The carcinoembryonic antigen assay in bronchogenic carcinoma.. Cancer.

[OCR_00619] Ellison M., Woodhouse D., Hillyard C., Dowsett M., Coombes R. C., Gilby E. D., Greenberg P. B., Neville A. M. (1975). Immunoreactive calcitonin production by human lung carcinoma cells in culture.. Br J Cancer.

[OCR_00625] Fischer J. A., Tobler P. H., Henke H., Tschopp F. A. (1983). Salmon and human calcitonin-like peptides coexist in the human thyroid and brain.. J Clin Endocrinol Metab.

[OCR_00631] Galan Galan F., Rogers R. M., Girgis S. I., Arnett T. R., Ravazzola M., Orci L., MacIntyre I. (1981). Immunochemical characterization and distribution of calcitonin in the lizard.. Acta Endocrinol (Copenh).

[OCR_00639] Galan Galan F., Rogers R. M., Girgis S. I., MacIntyre I. (1981). Immunoreactive calcitonin in the central nervous system of the pigeon.. Brain Res.

[OCR_00643] Girgis S. I., Galan F. G., Arnett T. R., Rogers R. M., Bone Q., Ravazzola M., MacIntyre I. (1980). Immunoreactive human calcitonin-like molecule in the nervous systems of protochordates and a cyclostome, Myxine.. J Endocrinol.

[OCR_00654] Goltzman D., Potts J. T., Ridgway R. C., Maloof F. (1974). Calcitonin as a tumor marker. Use of the radioimmunoassay for calcitonin in the postoperative evaluation of patients with medullary thyroid carcinoma.. N Engl J Med.

[OCR_00649] Goltzman D., Tischler A. S. (1978). Characterization of the immunochemical forms of calcitonin released by a medullary thyroid carcinoma in tissue culture.. J Clin Invest.

[OCR_00661] Gropp C., Havemann K., Lehmann F. G. (1978). Carcinoembryonic antigen and ferritin in patients with lung cancer before and during therapy.. Cancer.

[OCR_00672] Gropp C., Havemann K., Pflüger K. H. (1980). Calcitonin als Tumormarker beim Bronchialkarzinom.. Dtsch Med Wochenschr.

[OCR_00667] Gropp C., Havemann K., Scheuer A. (1980). Ectopic hormones in lung cancer patients at diagnosis and during therapy.. Cancer.

[OCR_00683] Ham J., Ellison M. L., Lumsden J. (1980). Tumour calcitonin. Interaction with specific calcitonin receptors.. Biochem J.

[OCR_00688] Jacobs J. W., Lund P. K., Potts J. T., Bell N. H., Habener J. F. (1981). Procalcitonin is a glycoprotein.. J Biol Chem.

[OCR_00693] Luster W., Gropp C., Sostmann H., Kalbfleisch H., Havemann K. (1982). Demonstration of immunoreactive calcitonin in sera and tissues of lung cancer patients.. Eur J Cancer Clin Oncol.

[OCR_00705] Milhaud G., Calmette C., Taboulet J., Julienne A., Moukhtar M. S. (1974). Letter: Hypersecretion of calcitonin in neoplastic conditions.. Lancet.

[OCR_00718] Perez Cano R., Girgis S. I., Galan Galan F., MacIntyre I. (1982). Identification of both human and salmon calcitonin-like molecules in birds suggesting the existence of two calcitonin genes.. J Endocrinol.

[OCR_00712] Perez-Cano R., Galan Galan F., Girgis S. I., Arnett T. R., MacIntyre I. (1981). A human calcitonin-like molecule in the ultimobranchial body of the amphibia (Rana pipiens).. Experientia.

[OCR_00723] Roos B. A., Lindall A. W., Baylin S. B., O'Neil J. A., Frelinger A. L., Birnbaum R. S., Lambert P. W. (1980). Plasma immunoreactive calcitonin in lung cancer.. J Clin Endocrinol Metab.

[OCR_00728] Silva O. L., Becker K. L., Primack A., Doppman J., Snider R. H. (1974). Ectopic secretion of calcitonin by oat-cell carcinoma.. N Engl J Med.

[OCR_00733] Sizemore G. W., Hpeath H., Larson J. M. (1975). Immunochemical heterogeneity of calcitonin in plasma of patients with medullary thryoid carcinoma.. J Clin Invest.

[OCR_00737] Vincent R. G., Chu T. M. (1973). Carcinoembryonic antigen in patients with carcinoma of the lung.. J Thorac Cardiovasc Surg.

